# Advances in peripheral nerve reconstruction: Current clinical conduits, comparative outcomes, limitations, and future technological perspectives

**DOI:** 10.1016/j.bas.2026.105951

**Published:** 2026-01-27

**Authors:** Shimon Rochkind

**Affiliations:** a Tel-Aviv University, Faculty of Medicine, Israel; b Microsurgical Center for Peripheral Nerve Reconstruction, Assuta Medical Center, Israel

## Abstract

**Introduction:**

Peripheral nerve injuries remain a major clinical challenge, often resulting in persistent motor deficits, sensory loss, and neuropathic pain. When direct tension-free repair is not feasible, nerve reconstruction relies on autografts, processed allografts, or conduit-based strategies. Despite decades of innovation, outcomes remain inconsistent, particularly in motor nerves, long-gap defects, and complex traumatic environments.

**Research question:**

What are the relative clinical performances and limitations of currently available nerve repair strategies, and which emerging technologies may address their unmet biological shortcomings?

**Material and methods:**

This narrative review synthesizes clinical, experimental, and comparative evidence on autologous nerve grafts, processed nerve allografts, and biological or synthetic nerve conduits. Emphasis is placed on reported functional recovery, gap-length tolerance, complication profiles, and the influence of the injury microenvironment.

**Results:**

Autologous nerve grafting remains the benchmark for reliable regeneration but is limited by donor-site morbidity and graft availability. Processed nerve allografts preserve extracellular matrix architecture and demonstrate outcomes comparable to autografts for moderate-length defects, while avoiding harvest-related complications. In contrast, hollow biological and synthetic conduits show acceptable performance primarily in short sensory gaps, with declining efficacy in motor nerves, long defects, and inflammatory or scar-prone injuries.

**Discussion and conclusion:**

Current nerve repair technologies are fundamentally constrained by their passive structural design and limited ability to modulate the inhibitory post-injury microenvironment. Future progress is likely to depend on integrative platforms that combine structural guidance with active biological modulation, including bioactive and hydrogel-based strategies, to enhance regeneration in complex peripheral nerve injuries.

## Highlights

• Autografts remain the benchmark, but donor-site morbidity drives alternatives.
• Processed nerve allografts preserve ECM guidance and avoid harvest morbidity.
• Conduits are most reliable in short sensory gaps; results fall in motor/long gaps.
• Passive conduits cannot counter fibrosis and inflammation in complex trauma.
• Future repair may combine structural bridging with bioactive hydrogel modulation.

## 1 Background

Peripheral nerve injuries continue to represent a significant challenge in neurosurgery and reconstructive microsurgery, frequently resulting in persistent motor deficits, sensory disturbances, and chronic neuropathic pain – consequences well documented across clinical and experimental literature (Lans et al., 2023). Following axonal transection, the distal segment undergoes Wallerian degeneration, during which Schwann cells re-establish a permissive microenvironment for regeneration. This process, described in foundational anatomical and regenerative studies, remains essential for functional recovery (Stocco et al., 2024).

As a clinician and researcher working in the field of nerve regeneration for several decades, I believe the future of peripheral nerve repair lies in the application of biotechnology to create next-generation synthetic nerve substitutes based on bioactive hydrogels – what I define as "neurogels" (Rochkind et al., 2021). These platforms are designed not only to bridge nerve gaps structurally but to actively modulate the biochemical and cellular microenvironment – suppressing scarring, enhancing axonal guidance, and promoting functional recovery. The development and optimization of such neurogels have been the focus of my work for many years, representing a promising new paradigm in neuroregenerative surgery.

When tension-free end-to-end neurorrhaphy cannot be achieved, surgeons must rely on interposition grafts to bridge the defect. Autologous nerve grafting remains the benchmark because it provides a naturally biomimetic scaffold containing basal lamina tubes, living Schwann cells, and native extracellular matrix components (de Ruiter et al., 2009). However, despite these biological advantages, autografting is associated with substantial limitations, including donor-site morbidity, limited graft availability, sensory loss at the harvest site, and potential fascicular mismatch (Lans et al., 2023). These constraints have motivated decades of innovation in developing alternative bridging materials. Current clinical options include synthetic conduits, collagen-based conduits, and processed decellularized nerve allografts, each attempting to replicate aspects of autograft biology with varying degrees of success (Stocco et al., 2024; de Ruiter et al., 2009; Kornfeld et al., 2021). Despite expanding the reconstructive possibilities, their performance remains inconsistent – especially in mixed or motor nerves, long-gap repairs, and complex trauma such as ballistic or shrapnel injuries, where the local environment is often severely compromised (Lans et al., 2023; Kornfeld et al., 2021).

This review synthesizes the current clinical landscape of nerve conduits, compares their outcomes with autografts and processed allografts, identifies intrinsic limitations of existing technologies, and examines emerging future strategies with emphasis on platforms designed to actively modulate the regenerative microenvironment.

## 2 Current clinical nerve conduits

A wide spectrum of nerve conduits is currently available for clinical use, each designed to provide a protected structural path for axonal regeneration when direct repair is not feasible. These devices differ markedly in composition, internal architecture, biomechanical behavior, and degree of biological activity – variables that critically influence regenerative outcomes, as extensively reviewed by Stocco et al. (2024) and de Ruiter et al. (de Ruiter et al., 2009). Their most predictable performance is observed in short, clean sensory nerve gaps, whereas results deteriorate significantly in mixed, motor, and long-gap injuries (Lans et al., 2023).

### 2.1 Collagen-based conduits

Type I collagen conduits (e.g., **NeuraGen®**) are widely used because of their biocompatibility, predictable biodegradation, and ease of handling. These hollow tubes support early fibrin matrix formation and Schwann cell migration but provide minimal internal architecture. As documented by Stocco et al. (2024), their simple design limits their ability to maintain lumen patency, prevent collapse, or guide axons with precision.

The next-generation **NeuraGen® 3D**, incorporating a chondroitin-6-sulfate (C6S)-infused 3D inner matrix, demonstrated superior axon counts, nerve fiber density, CMAP recovery, and muscle outcomes compared with hollow conduits in preclinical studies (Lee et al., 2012). However, even these improvements have not translated into reliable performance in defects longer than 20–30 mm or in mixed motor nerves (Stocco et al., 2024).

Collagen conduits therefore remain appropriate mainly for **short sensory nerve gaps** with well-defined, non-scarred margins.

Collagen conduits are among the most widely used clinically due to their biocompatibility, biodegradability, and ability to provide a permissive scaffold for Schwann cell migration.

Nevertheless, collagen conduits remain limited to small-diameter nerves and defects generally ≤30 mm, as recognized in both preclinical and clinical studies. Hollow conduits lack structural reinforcement and often collapse in larger gaps, reducing the regenerative potential.

### 2.2 Synthetic polymer conduits

Synthetic biodegradable conduits – constructed from materials such as **polyglycolic acid (PGA), polycaprolactone (PCL),** and **polylactide-co-glycolide (PLGA) –** offer mechanical strength and customizable degradation profiles. Widely referenced examples include: **Neurotube® (PGA), Neurolac® (PCL), SaluBridge®.** These conduits can be engineered for porosity, stiffness, and permeability, but they often elicit foreign-body inflammatory responses and degrade unpredictably, leading to fragmentation or compression of regenerating fibers (de Ruiter et al., 2009). Importantly, they lack neurotrophic or cellular support and therefore provide only passive mechanical guidance. Clinical outcomes consistently show that synthetic conduits perform adequately **only in short sensory gaps**, with rapidly declining effectiveness as gap length or injury complexity increases (Lans et al., 2023).

### 2.3 Processed nerve allografts

Processed decellularized nerve allografts, most notably **Avance® Nerve Graft,** preserve the extracellular matrix architecture while removing immunogenic cellular components. These scaffolds maintain the intrinsic basal lamina microtubes that guide axonal regeneration more effectively than hollow conduits, as demonstrated by Kornfeld et al. (2021). Multiple clinical studies show that processed allografts can achieve meaningful sensory and mixed-nerve recovery comparable to autografts for defects up to **70 mm**, without donor-site morbidity (Lans et al., 2023).

Yet several limitations remain:

• loss of viable Schwann cells during decellularization,
• reduced performance in motor-dominant nerves,
• diminished efficacy in long (>70 mm) or heavily scarred injuries,
• vulnerability to poor outcomes in ballistic/shrapnel trauma where inflammation dominates (Kornfeld et al., 2021).

Despite these challenges, processed allografts currently represent the most clinically validated alternative to autografting.

Overall: **Autografts** and **processed allografts** consistently outperform conduits in mixed or motor nerves and in long defects (Lans et al., 2023; Kornfeld et al., 2021). **Conduits** show the greatest variability in outcomes and the highest rates of persistent neuropathic symptoms or neuroma formation (Lans et al., 2023). **Complex trauma,** such as ballistic or shrapnel injuries, unmask the limitations of passive conduits, which cannot modulate fibrosis, inflammation, or the biochemical microenvironment (Stocco et al., 2024; Kornfeld et al., 2021). These shortcomings underscore the need for next-generation nerve repair platforms that integrate structural guidance with active biological modulation.

## 3 Autograft vs Allograft vs conduits: comparative evidence

A substantial body of clinical research has compared outcomes across autografts, processed allografts, and various conduit systems. The most comprehensive synthesis to date is the 2023 systematic review and meta-analysis by Lans et al. (2023), which evaluated 1559 nerve repairs across 35 clinical studies, spanning nerve gaps from >5 mm to 70 mm. This meta-analysis provides the highest-level comparative evidence currently available.

**Meaningful Recovery (MR):** Lans et al. (2023) demonstrated that autografts and processed allografts achieve statistically comparable rates of meaningful sensory and motor recovery, defined as ≥ S3/M3 on the Medical Research Council Classification system (Lans et al., 2023). The pooled MR rates were:

• Allograft: 87.1 %
• Autograft: 81.6 %
• Conduits: 62.2 % (significantly inferior; *p* < 0.05)

These findings confirm that, although autografts remain the gold standard, allografts perform equivalently across a wide range of defect lengths, while conduits are notably less effective beyond very short sensory repairs.

**Short-Gap vs Long-Gap Outcomes:** The distinction between short-gap (>5 to ≤30 mm) and long-gap repairs (>30 to ≤70 mm) is crucial. In short sensory gaps, autografts and allografts again showed equivalent MR rates, whereas conduit outcomes dropped significantly (MR ≈ 60 %) (Lans et al., 2023). In long gaps, conduits are inadequate, while allografts maintained meaningful recovery approaching autograft levels, although performance decreased for motor-dominant nerves or injuries with extensive scarring (Lans et al., 2023; Kornfeld et al., 2021).

### 3.1 Complications

• Conduits had notably higher rates of persistent pain, sensory disturbances, and symptomatic neuroma formation (Lans et al., 2023).
• Allografts demonstrated complication rates similar to autografts but without donor-site morbidity (Lans et al., 2023; Kornfeld et al., 2021).
• Autograft harvest remains associated with sensory deficits, donor nerve pain, potential wound complications, and reduced satisfaction.

**Economic Considerations:** According to the cost meta-analysis (Lans et al., 2023), allograft procedures did not incur higher expenses than autografts once total hospital costs, surgery time, and the morbidity associated with donor nerve harvesting were taken into consideration.

### 3.2 Implications for clinical practice

These data suggest a clear hierarchy of effectiveness:

• Autografts → gold standard, particularly for long gaps, motor nerves, and complex trauma
• Allografts → validated alternative with comparable outcomes up to 70 mm and lower morbidity
• Conduits → useful primarily for short (<20–30 mm) sensory nerve gaps

This evidence strongly supports the ongoing shift away from hollow conduits and toward biologically active or biologically derived scaffolds.

**Limitations of Existing Technologies:** Despite decades of development, currently available nerve repair technologies share several fundamental limitations that constrain their effectiveness, particularly outside short sensory gaps. These limitations stem from material properties, biological inactivity, and inadequate interaction with the complex microenvironment of peripheral nerve injury.

### 3.3 Insufficient biological activity and lack of neurotrophic support

Most clinically used conduits – whether collagen-based or synthetic – function primarily as passive mechanical guides. They do not replicate the biological richness of autografts, which provide basal lamina scaffolds, neurotrophic factors, and Schwann cell-driven guidance.

As highlighted by Stocco et al. (2024), the absence of topographically meaningful internal architecture and the lack of cell-derived cues result in suboptimal axonal organization and diminished regenerative capacity. Similarly, de Ruiter et al. (de Ruiter et al., 2009) showed that hollow conduits fail to sustain the early fibrin matrix beyond very short gaps, causing disorganized sprouting and misdirection.

**Limited Gap Length Tolerance:** The clinical performance of conduits declines sharply as gap length increases. According to the systematic review by Lans et al. (2023), conduits demonstrate acceptable outcomes only in sensory gaps up to 20–30 mm, with significant deterioration beyond this range.

This limitation is attributed to:

• collapse of the hollow lumen
• inadequate maintenance of a stable fibrin scaffold
• insufficient Schwann cell migration across longer distances

In contrast, processed allografts retain structural integrity up to 70 mm, yet even they show declining performance in very long gaps due to loss of viable Schwann cells and progressive matrix degradation (Kornfeld et al., 2021).

### 3.4 Susceptibility to fibrosis, scar formation, and inflammatory microenvironment

One of the major biological barriers to regeneration is fibrosis.

Hollow conduits permit infiltration of fibroblasts through their porous walls, leading to luminal scarring, which interrupts axonal continuity – a phenomenon described in detail by Stocco et al. (2024) and de Ruiter et al. (de Ruiter et al., 2009).

Furthermore, Lans et al. (2023) demonstrated that patients receiving conduit repairs exhibit higher rates of persistent pain and sensory disturbances, likely reflecting pathological scarring, neuroma formation, or chronic inflammation.

These issues are exacerbated in complex trauma – such as ballistic and shrapnel injuries – where the tissue microenvironment contains necrotic debris, metal fragments, and chronic inflammatory cells.

**Poor Performance in Motor and Mixed Nerves:** Motor axons have more demanding biological requirements than sensory fibers. They depend on:

• timely Schwann cell support,
• precise fascicular alignment,
• adequate neurotrophic signaling.
  Because conduits lack these elements, outcomes in motor nerves are predictably weaker.
Kornfeld et al. (2021) specifically note that allografts – despite outperforming conduits – also decline in effectiveness in motor-dominant repairs when gap length increases or local scarring is significant.

### 3.5 Static and non-adaptive scaffold architecture

A key conceptual limitation is that current conduits are mechanically static: they do not adjust to changes in the regenerating tissue, do not modulate inflammation, and do not actively counteract glial or fibrotic scarring. Advanced designs (microgrooved, porous, multichannel) may mitigate some of these shortcomings, but as Stocco et al. (2024) emphasize, manufacturing complexity and incomplete biological integration still limit their clinical applicability.

## 4 Future perspectives: next-generation platforms for peripheral nerve reconstruction

Despite the substantial progress achieved with autografts, processed allografts, and current-generation conduits, the field of peripheral nerve reconstruction is on the threshold of a more biologically sophisticated era. Emerging strategies increasingly focus not only on providing structural continuity but also on modulating the biochemical and cellular microenvironment that promotes regeneration. Several converging research directions hold promise for overcoming the inherent limitations of existing approaches.

1. **Biomimetic and Multichannel Conduits:** Advances in fabrication technologies – including electrospinning, photolithography, and 3D printing – have enabled the creation of conduits that more closely reproduce the fascicular architecture of peripheral nerves.

Microgrooved, multichannel, and anisotropic luminal designs have been shown to:

• enhance Schwann cell alignment,
• reduce axonal dispersion,
• improve directional guidance,
• limit mismatched reinnervation.

Stocco et al. (2024) emphasized that topographically patterned luminal surfaces can significantly influence neurite outgrowth, but also noted that manufacturing complexity and scalability continue to limit clinical translation. Similarly, de Ruiter et al. (de Ruiter et al., 2009) demonstrated that multichannel constructs improve regenerative organization experimentally, though their mechanical fragility and reduced internal cross-sectional area remain unresolved barriers.

2. **Hydrogel-Based Regenerative Matrices:** Injectable hydrogels – including fibrin, collagen, hyaluronic acid, self-assembling peptides, and PEG-based systems – represent one of the most dynamic research areas in nerve engineering. Hydrogels can:

• conform to irregular or complex defects,
• support early fibrin matrix formation,
• sustain Schwann cell migration,
• deliver biomolecules or support cells,
• maintain a hydrated, permissive microenvironment.

These features mitigate several weaknesses of hollow conduits, particularly their inability to adapt to the injury landscape. As de Ruiter et al. (de Ruiter et al., 2009) noted, hydrogels allow direct modulation of the intraneural milieu, an advantage in cases where the wound bed is heterogeneous, scarred, or distorted by trauma. Hydrogels are also being explored as fillers inside conduits, potentially enhancing performance without altering the conduit's structural role.

**Cell-Enriched and Cell-Interactive Constructs:** The use of autologous Schwann cells, Schwann-like cells derived from stem cell populations, or genetically modified glial cells has repeatedly shown improved regeneration in preclinical studies. These constructs offer:

• enhanced neurotrophic support
• improved myelination capacity
• biochemical guidance unavailable in decellularized scaffolds

However, cell-enriched constructs face major translational challenges, including:

• lengthy cell expansion times
• high production costs
• sterility and regulatory considerations
• complex logistics unsuited to acute trauma settings

Kornfeld et al. (2021) stress that although allogenic tissue engineering holds promise, its wide-scale clinical application is constrained by these limitations.

4. Controlled Delivery of Trophic and Immunomodulatory Factors

Another promising direction involves scaffolds engineered for sustained or spatially controlled release of bioactive molecules such as:

• neurotrophic factors (NGF, BDNF, GDNF)
• angiogenic signals (VEGF)
• anti-inflammatory cytokines
• extracellular matrix–modifying enzymes

Such systems attempt to reproduce the dynamic trophic gradients found in autografts. De Ruiter et al. (de Ruiter et al., 2009) noted that binding factors to matrix components or encapsulating them in microspheres can prolong local signaling while preventing rapid diffusion. However, the risk of aberrant axonal sprouting, unpredictable biodistribution, and regulatory complexity continue to limit clinical adoption.

5. **Conductive and Electrostimulative Scaffolds:** Bioelectronic approaches – including conductive polymers (polypyrrole, PEDOT), graphene-based composites, and piezoelectric scaffolds – seek to leverage the known benefits of electrical stimulation on axonal growth. Early data shows improved neurite extension and Schwann cell activation in vitro, but long-term safety, biodegradation profiles, and durability remain insufficiently understood, preventing clinical deployment.
6. **Microenvironment-Modulating and Antigliotic Platforms (Including AGRG)**

A central limitation of current conduits is their inability to address the injury-induced inhibitory microenvironment, characterized by fibrosis, extracellular matrix dysregulation, and scarring. A growing class of emerging technologies focuses on modifying these pathological biochemical conditions.

These include:

• matrices that reduce deposition of inhibitory chondroitin sulfate proteoglycans,
• scaffolds releasing anti-fibrotic or anti-inflammatory factors,
• hydrogels designed to limit glial scar formation,
• enzyme-functionalized constructs targeting scar-associated molecules

Within this broader category, regenerative hydrogels – such as the Antigliotic Guiding Regenerative Gel (AGRG) (Rochkind et al., 2021) – represent an example of platforms designed to reduce scar formation and promote a more permissive microenvironment for axonal regeneration. AGRG is a promising new biomaterial for nerve repair, engineered to actively modulate the inhibitory post-injury microenvironment. As part of the broader class of microenvironment-modulating technologies that may function as adjuncts to autografts, allografts, or advanced conduits, AGRG represents a next-generation, clinically oriented platform with strong translational potential. Notably, AGRG has progressed beyond early-stage development and is already advancing through the FDA regulatory pathway.

## 5 Conclusion

Despite continuous refinements in microsurgical technique and biomaterial science, peripheral nerve reconstruction remains one of the most biologically demanding areas in reconstructive surgery. The current clinical hierarchy – where autologous nerve grafting remains the benchmark, processed allografts represent the most validated alternative, and conduits retain value primarily in short sensory defects – reflects the limitations of existing technologies rather than the ultimate potential of nerve repair. Autografts continue to deliver the most reliable outcomes because they uniquely provide structural, cellular, and trophic support necessary for coordinated axonal regeneration. Processed allografts have significantly narrowed the performance gap by preserving extracellular matrix architecture while eliminating donor-site morbidity, achieving meaningful recovery across defects up to 70 mm, although their efficacy declines in motor-dominant or severely scarred injuries (Lans et al., 2023; Kornfeld et al., 2021). By contrast, hollow biological and synthetic conduits – while clinically useful – remain fundamentally constrained by their passive, non-interactive architecture, a limitation repeatedly emphasized across experimental and clinical literature (Stocco et al., 2024; de Ruiter et al., 2009).

The future of peripheral nerve reconstruction will likely be shaped not by a single technology, but by integrative platforms that combine structural guidance with microenvironmental modulation. Promising strategies – including biomimetic multichannel conduits, bioactive hydrogels, cell-enriched constructs, controlled-release systems, and antigliotic matrices – seek to address the biological obstacles that currently limit regeneration, particularly in long gaps or complex trauma (Stocco et al., 2024; de Ruiter et al., 2009; Kornfeld et al., 2021). Among these emerging approaches, some antigliotic hydrogel-based platforms, such as AGRG (Rochkind et al., 2021), have already entered early stages of regulatory evaluation, reflecting a broader trend toward clinically oriented materials designed to influence the local injury environment.

As these next-generation technologies advance, rigorous translational research and well-designed clinical studies will be essential to determine how they can be incorporated into modern algorithms of nerve repair. Ultimately, the convergence of biomaterials engineering, cellular neuroscience, and microsurgical innovation holds the potential to redefine the therapeutic landscape of peripheral nerve injury – moving the field beyond mechanical bridging and toward solutions that dynamically interact with, and actively support, the regenerative process.

## Declaration of competing interest

The authors declare that they have no known competing financial interests or personal relationships that could have appeared to influence the work reported in this paper.
